# Consequences of Vitamin A Deficiency: Immunoglobulin Dysregulation, Squamous Cell Metaplasia, Infectious Disease, and Death

**DOI:** 10.3390/ijms21155570

**Published:** 2020-08-04

**Authors:** Sherri L. Surman, Rhiannon R. Penkert, Robert E. Sealy, Bart G. Jones, Tony N. Marion, Peter Vogel, Julia L. Hurwitz

**Affiliations:** 1 Department of Infectious Diseases, St. Jude Children’s Research Hospital, Memphis, TN 38105, USA; sherri.surman@stjude.org (S.L.S.); rhiannon.penkert@stjude.org (R.R.P.); bob.sealy@stjude.org (R.E.S.); bart.jones@stjude.org (B.G.J.); 2 Department of Microbiology, Immunology and Biochemistry, University of Tennessee Health Science Center, Memphis, TN 38163, USA; tmarion@uthsc.edu; 3 Department of Pathology, St. Jude Children’s Research Hospital, Memphis, TN 38105, USA; peter.vogel@stjude.org

**Keywords:** vitamin A deficiency, squamous cell metaplasia, abnormal cytokines, abnormal immunoglobulins, infections

## Abstract

Vitamin A is an important regulator of immune protection, but it is often overlooked in studies of infectious disease. Vitamin A binds an array of nuclear receptors (e.g., retinoic acid receptor, peroxisome proliferator-activated receptor, retinoid X receptor) and influences the barrier and immune cells responsible for pathogen control. Children and adults in developed and developing countries are often vitamin A-deficient or insufficient, characteristics associated with poor health outcomes. To gain a better understanding of the protective mechanisms influenced by vitamin A, we examined immune factors and epithelial barriers in vitamin A deficient (VAD) mice, vitamin D deficient (VDD) mice, double deficient (VAD+VDD) mice, and mice on a vitamin-replete diet (controls). Some mice received insults, including intraperitoneal injections with complete and incomplete Freund’s adjuvant (emulsified with PBS alone or with DNA + Fus-1 peptide) or intranasal inoculations with Sendai virus (SeV). Both before and after insults, the VAD and VAD+VDD mice exhibited abnormal serum immunoglobulin isotypes (e.g., elevated IgG2b levels, particularly in males) and cytokine/chemokine patterns (e.g., elevated eotaxin). Even without insult, when the VAD and VAD+VDD mice reached 3–6 months of age, they frequently exhibited opportunistic ascending bacterial urinary tract infections. There were high frequencies of nephropathy (squamous cell hyperplasia of the renal urothelium, renal scarring, and ascending pyelonephritis) and death in the VAD and VAD+VDD mice. When younger VAD mice were infected with SeV, the predominant lesion was squamous cell metaplasia of respiratory epithelium in lungs and bronchioles. Results highlight a critical role for vitamin A in the maintenance of healthy immune responses, epithelial cell integrity, and pathogen control.

## 1. Introduction

Vitamin A and D deficiencies have long been recognized as dietary concerns in developing countries. For example, the increased morbidity and mortality caused by measles infections have been associated with low vitamin A levels [1]. The WHO and other organizations support vitamin supplementation programs to correct vitamin A deficits and improve pediatric health [2,3,4,5].

Over the past decade, diets in the developed world have changed considerably. Vitamin A and D deficits are now prevalent among low-income families, but because clinical tests for serum retinol are not routine, the precise frequencies of vitamin deficiencies and insufficiencies are unknown. Our study of vitamins A and D among individuals in Memphis, Tennessee, using retinol binding protein (RBP) as a surrogate for blood retinol showed that the majority of tested individuals were deficient or insufficient for vitamin D, and approximately half of the individuals were deficient or insufficient for vitamin A [6]. Some of the tested individuals may have been experiencing infections, a situation that could reduce the RBP levels, but we observed low levels in individuals without frank indications of disease. The low vitamin levels in Memphis were further associated with poor outcomes among children hospitalized with respiratory syncytial virus (RSV) or human metapneumovirus respiratory infections [7]. 

The physiological effects of vitamins A and D are vast in number. The activities influenced by vitamins A and D among structural and immune cells include proliferation, cytokine/chemokine production, and migration [8,9,10]. Among their many functions, vitamin A and D metabolites serve as ligands for nuclear receptors. Heterodimeric receptors include the retinoic acid receptor-retinoid X receptor (RAR-RXR), the peroxisome proliferator-activated receptor-RXR (PPAR -RXR), and the vitamin D receptor-RXR (VDR-RXR) [11,12,13]. The vitamin A metabolite all-trans retinoic acid binds RAR or PPARβ/δ, whereas 9-*cis*-retinoic acid binds RXR. Influenced by their binding to ligands, receptors bind DNA response elements (e.g., the retinoic acid response element, RARE). They then serve as transcription factors to regulate gene expression [14]. We have recently found that nuclear receptors bind key switch (S) sites, promoters, and enhancers in the immunoglobulin heavy chain gene locus, suggesting a direct mechanism by which vitamins and other nuclear hormones might regulate antibody levels. If/when nuclear receptors contribute to enhanceosomes and switchosomes within regulatory regions of the immunoglobulin loci, they may influence class switch recombination (CSR) and thereby instruct the production of IgM, IgG, IgE, and IgA by activated B cells [15,16,17,18,19,20]. 

To better understand the influences of vitamin deficiencies, we examined vitamin A deficient (VAD), vitamin D deficient (VDD), and double deficient (VAD+VDD) mice compared to mice on a vitamin replete diet (controls) for immunoglobulin isotypes, serum factors, and epithelial cell barrier integrity. Mice received various insults, including intraperitoneal (IP) injections of complete and incomplete Freund’s adjuvant administered with or without a DNA-Fus 1 peptide mixture [21,22,23,24]. Additional insults included intranasal (IN) inoculations with a murine respiratory pathogen, Sendai virus (SeV). Results demonstrated abnormalities in the VAD and VAD+VDD mice, both before and after insults. The VAD and VAD+VDD mice suffered abnormal immunoglobulin isotype and cytokine profiles, infectious disease, and squamous cell metaplasia at mucosal sites. The VAD and VAD+VDD mice suffered frequent death, unlike their VDD and control counterparts.

## 2. Results

### 2.1. Abnormal Immunoglobulin and Cytokine/Chemokine Profiles in VAD and VAD+VDD Mice

Pregnant C57BL/6 mice were placed on test diets at 5 days gestation, and upon birth the pups were maintained on the same diets until adulthood and throughout experimentation. There were four diets: VAD, VDD, VAD+VDD, and the control. Experiments were begun when offspring reached adulthood (6–8 weeks of age). Test mice received consecutive intraperitoneal (IP) injections with complete and incomplete Freund’s adjuvant emulsified with PBS alone (termed CFA) or DNA + Fus-1 peptide (termed CFA/DNA/PEP, see Materials and Methods) as insults [22,23,24]. Additional mice received PBS only.

**Figure 1:**
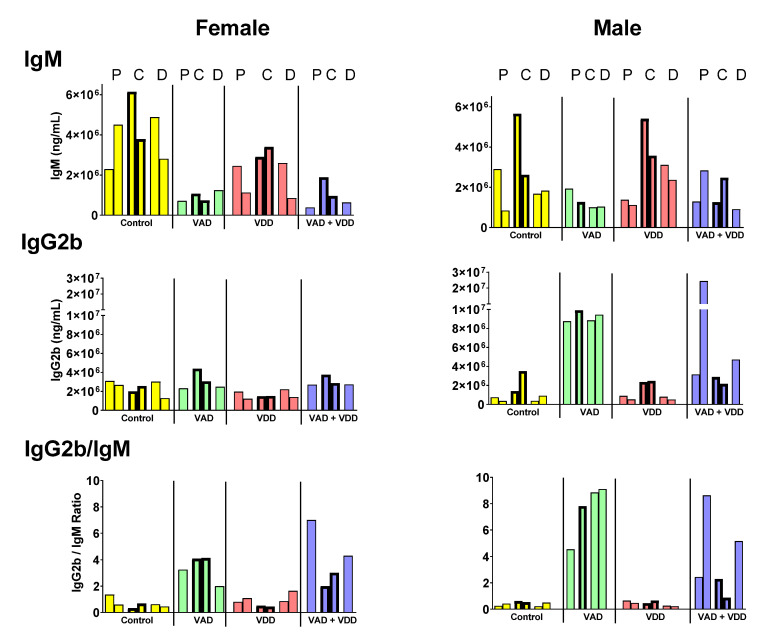
Serum immunoglobulin isotypes in animal groups. Isotypes and isotype ratios are shown for mice that were injected with PBS, complete and incomplete Freund’s adjuvant (emulsified with PBS alone [termed CFA] or DNA + Fus-1 peptide [termed CFA/DNA/PEP]). Mice received one prime and one booster injection and were bled 6 days after the booster. Data from mice injected with PBS only were described previously [16]. Bars represent the total immunoglobulin levels (ng/mL) for individual mice (Yellow **=** control mice; green **=** vitamin A deficient [VAD] mice; pink **=** vitamin D deficient [VDD] mice; blue **=** double deficient [VAD+VDD] mice). For each group of females or males, the bars represent mice injected with PBS (P, thin borders) CFA (C, thick borders), or CFA/DNA/PEP (D, thin borders). When the mice received similar injections, the bars were placed side-by-side with no intervening space.

To identify diet-associated changes, we examined the mice for their total serum immunoglobulins (Figure 1). We previously reported that in PBS-injected mice, the VAD and VAD+VDD diets affected immunoglobulin isotypes [16].

In Figure 1, we show representative results from mice injected with PBS, CFA, or CFA/DNA/PEP. In the VAD (and VAD+VDD) mice, there were lower IgM levels compared to animals on the control diet, particularly among females (*p* < 0.01 for VAD females compared to the control females, Mann Whitney test). These differences were evident in animals regardless of the injection type. In male VAD mice, the IgG2b levels were significantly higher than the levels in control males (*p* < 0.01) or VAD females (*p* < 0.05). In both males and females, the VAD (and VAD+VDD) mice had higher IgG2b/IgM ratios compared to the controls (*p* < 0.01 for VAD females compared to the control females; *p* < 0.01 for VAD males compared to the control males). These features were dependent on diets and not on the type of injections that mice received.

**Figure 2:**
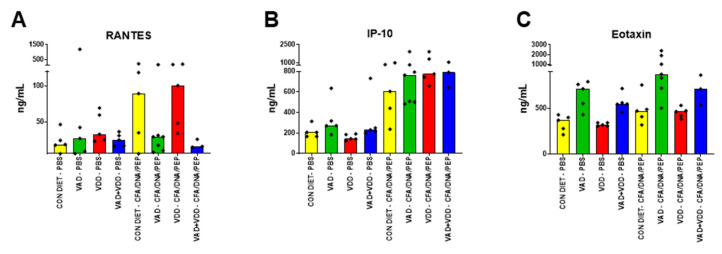
Changes in the blood cytokines/chemokines based on diets. Results are shown from a representative experiment. Mice received consecutive injections, either with PBS or with complete and incomplete Freund’s adjuvant emulsified with DNA + Fus-1 peptide (CFA/DNA/PEP). Mice were primed and boosted and sampled approximately 3–4 weeks after the booster. Results are shown for regulated upon activation, normal T cell expressed and secreted (RANTES, (**A**)), interferon γ-inducible protein 10 (IP-10, (**B**)), and eotaxin (**C**). Yellow **=** control mice (mice on the vitamin-replete, control diet, “CON DIET”); green **=** vitamin A deficient (VAD) mice; red **=** vitamin D deficient (VDD) mice; blue **=** double deficient (VAD+VDD) mice. Each symbol represents a different mouse, with medians indicated by bar heights.

We additionally examined the cytokine/chemokine levels known to be associated with inflammation and cell repair [25,26,27,28]. In each of two experiments, we found that the cytokines/chemokines were altered both by the diets and by the CFA/DNA/PEP insults (results of one representative experiment are shown in Figure 2). When the “Combined VAD” mice (including VAD and VAD+VDD mice) were compared to the Non-VAD mice (including VDD and control mice) after the CFA/DNA/PEP injections, the regulated upon activation, normal T cell expressed and secreted (RANTES) chemokine concentrations were significantly reduced (Figure 2A, *p* = 0.0083, Mann Whitney tests). IL-1α and IL-5 were also reduced in the “Combined VAD” mice compared to the Non-VAD mice. 

In contrast, in the PBS-injected animals the “Combined VAD” mice produced higher levels of interferon γ-inducible protein 10 (IP-10) compared to the Non-VAD mice (Figure 2B, *p* = 0.0039, Mann Whitney Test). In the animals injected with CFA/DNA/PEP, the IP-10 levels increased in all the diet groups. The “Combined VAD” mice produced higher levels of eotaxin compared to the Non-VAD mice, both following the PBS injections (Figure 2C, *p* < 0.0001) and the CFA/DNA/PEP injections (Figure 2C, *p* = 0.0002, Mann Whitney). 

### 2.2. Squamous Cell Metaplasia of the Renal Pelvis and Ascending Bacterial Urinary Tract Infections (UTI) in VAD and VAD+VDD Mice 

The PBS, CFA, or CFA/DNA/PEP-injected mice on the four diets were examined for histopathologic lesions. Assessments were performed when the animals had reached 3–6 months of age. We found that many VAD and VAD+VDD mice developed squamous cell metaplasia of the renal pelvis, with ascending bacterial UTIs and renal scarring.

**Figure 3:**
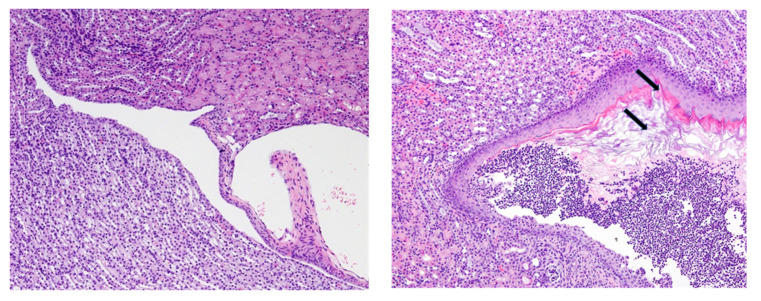
Histopathologic analyses of epithelial changes and inflammation in the renal pelvis and cortex of mice on vitamin A deficient (VAD) diets. Mice were injected with complete Freund’s adjuvant followed by incomplete Freund’s adjuvant approximately 3 weeks later and sacrificed approximately 3–4 weeks after the second injection. Representative tissue sections are shown (10× magnification). (Left) a mouse on the normal control diet. The urothelium lining the renal pelvis and fornices consisted of a thin layer (1–3 cells thick) of polygonal cells with clearly defined nuclei. (Right) in a VAD mouse, there was marked thickening of the squamous epithelium, with the cornification of upper layers (top arrow) and the extensive sloughing of cornified cells into the lumen of the renal pelvis (bottom arrow).

Disease was frequent regardless of injection (PBS, CFA, or CFA/DNA/PEP), suggesting that disease was a consequence of the diets and not the insults. In mice on the control diet, the urothelium lining of the renal pelvis and fornices consisted of a thin layer (1–3 cells thick) of polygonal cells with clearly defined nuclei (Figure 3, left image). In contrast, the urothelium in the VAD mice was a markedly thickened highly stratified squamous epithelium, with cornification of the upper layers and extensive sloughing of cornified cells into the lumen of the renal pelvis (Figure 3, right image). The urothelium in the VDD mice was normal, whereas the renal pelvis lesions in the VAD+VDD mice were indistinguishable from those of the VAD mice, being characterized by the accumulation of abundant desquamated keratinized squamous epithelium and cell debris in the lumen, with neutrophilic inflammation and bacteria.

Lesions in the renal cortex were also limited to mice with VAD or VAD+VDD. In contrast to the normal arrangement of renal tubules and glomeruli in mice on normal diets and VDD mice, there was frequent, chronic inflammation and fibrosis extending into the adjacent medulla and cortex in both the VAD and VAD+VDD mice, with extensive scarring of the renal parenchyma in some cases. Overall, these renal lesions were indicative of an ascending pyelonephritis. Nephropathies and ascending bacterial UTIs were common in the VAD and VAD+VDD mice. 

When full body analyses were performed, inflammation was also observed in tissues other than the kidneys, and in some cases bacteria were identified in the lungs, heart, liver, kidney, intestine, brain, and spinal column. 

The combined results from more than one experiment are shown in Table 1 (note that the experiments were not equally balanced for the numbers of animals per group). The frequencies of nephropathies in the “Combined VAD” and Non-VAD mice were significantly different (*p* < 0.001, Fisher’s exact test).

Deaths were frequent among the male and female VAD and VAD+VDD mice that had reached 3–6 months of age. Deaths occurred among mice that received any of the injections (PBS, CFA, or CFA/DNA/PEP). In one independent representative experiment, the deaths were 26/39 among the VAD mice, 14/22 among the VAD + VDD mice, 0/22 among the VDD mice, and 0/24 among the control mice. The differences between the “Combined VAD” and Non-VAD mice were significant (*p* < 0.0001, Fisher’s exact test).

### 2.3. Abnormal Epithelial Cell Barriers in the Airways of VAD Mice Infected with a Respiratory Virus 

We examined a second epithelial barrier, the respiratory tract lining. In this case, we evaluated the airways of young adult unmanipulated VAD and control mice, as well as mice that had been infected with SeV and sacrificed 22–29 days later (in some cases, the mice received a second dose of SeV two days prior to sacrifice, but this did not affect the outcome described below).

Upper respiratory tract lesions were not observed in the uninfected naïve or VAD control mice. However, in the SeV-infected VAD mice, there was widespread squamous metaplasia over the anterior turbinates, with patchy mild squamous metaplasia and keratinization in the larynx and trachea. 

**Figure 4:**
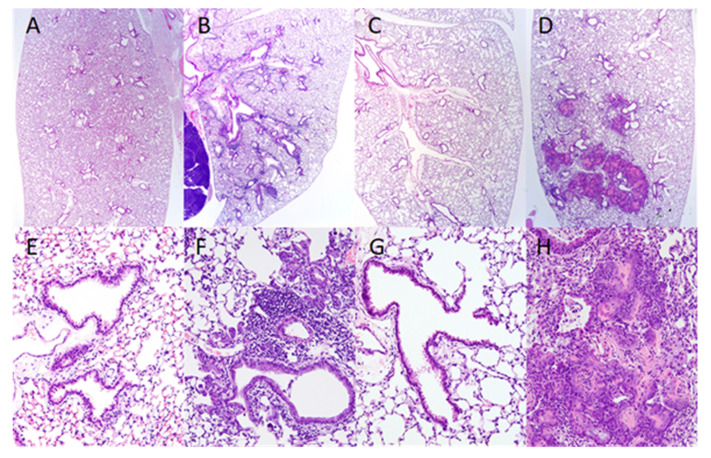
Histopathologic analysis of the epithelial changes and inflammation in the lungs of mice on control or vitamin A deficient (VAD) diets with or without Sendai virus (SeV) infections. Representative tissue sections are shown. (**A**) Normal lower respiratory tract in an uninfected mouse on the control diet. (**B**) Widespread perivascular/peribronchiolar inflammatory cell infiltrates and the mild thickening of the alveolar septa following SeV infection (after two SeV exposures, see Materials and Methods) in a mouse on a control diet. (**C**) Normal lower respiratory tract epithelium in an uninfected mouse on the VAD diet. (**D**) Reduced inflammation but extensive squamous metaplasia of the bronchiolar and alveolar epithelium in an SeV-infected mouse (after two SeV exposures) on the VAD diet. Higher magnification photos (**E–H,** 20× magnification) of pulmonary changes after the same treatments as (**A–D,** 2× magnification). (**E**) Normal terminal airway epithelium of an uninfected mouse on the vitamin-replete, control diet. (**F**) Perivascular/peribronchiolar lymphocytic infiltrates and mild accumulations of cell debris and macrophages after SeV infection in a mouse on the control diet. Small foci of alveolar bronchiolization are present (top right quadrant). (**G**) Absence of pulmonary lesions in an uninfected VAD mouse. (**H**) In an SeV-infected VAD mouse, inflammatory cell infiltrates are markedly reduced, but there is extensive squamous metaplasia and there are accumulations of keratinized epithelium, blocking the terminal airways and adjacent alveoli.

Lower respiratory tract lesions were not observed in the lungs of uninfected mice on normal diets (Figure 4A) or uninfected VAD mice (Figure 4C). In the SeV-infected mice on normal control diets, there were widespread perivascular/peribronchiolar inflammatory cell infiltrates and a mild thickening of the alveolar septa in affected areas (Figure 4B). In contrast, the lesions in SeV-infected VAD mice were characterized by reduced inflammatory cell infiltrates but extensive squamous metaplasia of the bronchiolar and alveolar epithelium (Figure 4D). The squamous metaplasia of the airway epithelium was characterized by the replacement of the normal single layer cuboidal and low columnar bronchiolar epithelium and flattened alveolar cells with multiple layers of keratinized squamous epithelium. 

At higher magnifications, inflammatory cell infiltrates and epithelial changes were not present around the terminal airways of either uninfected control mice (Figure 4E) or uninfected VAD mice (Figure 4G). The SeV-infected mice on control diets showed perivascular/peribronchiolar lymphocytic infiltrates and mild accumulations of cell debris and macrophages in the peribronchiolar alveoli (Figure 4F). There were a few foci of alveolar bronchiolization in some lungs (Figure 4F). In the virus-infected VAD mice, pulmonary lesions were also centered on bronchioles, but inflammatory cell infiltrates in the VAD mice were reduced in comparison to the infected controls. The squamous metaplasia and accumulations of keratinized and cornified epithelial cells expanded and blocked the terminal airways and adjacent alveoli (Figure 4H) in affected areas. These lesions frequently coalesced with similar lesions affecting adjacent bronchioles and could involve extensive areas of the lung parenchyma.

In one set of analyses, among mice infected with SeV, the squamous metaplasia of the distal bronchiolar and proximal alveolar epithelium was observed in 5 out of 11 VAD mice and 0 out of 12 mice on the control diet (*p* = 0.014, Fisher’s Exact test). Taken together, our findings indicate that two different conditions, (i) VAD and (ii) a direct insult to the epithelia (in this case, virus-induced), were required in combination to induce squamous metaplasia in the lungs of young adult animals. 

## 3. Discussion

Here, we describe immune and barrier parameters among VAD, VDD, VAD+VDD, and control mice. The insults used in our study included injections with adjuvants administered with or without DNA-Fus 1 peptide mixtures, previously shown to induce inflammatory responses [21,22,23,24]. Infections with SeV, a common murine respiratory pathogen, were also tested. We found that regardless of insult, the VAD and VAD+VDD mice frequently suffered squamous metaplasia, accompanied by ascending bacterial infections in the renal pelvis and death after 3–6 months of age. Squamous metaplasia was also observed in the airways of younger VAD mice infected with SeV. Abnormalities in the epithelial barriers were not surprising, given that epithelial cells are known to produce RAR-RXR, retinoic acid binding proteins [29], and retinaldehyde dehydrogenase ALDH1A [30] (used for the metabolism of retinal to retinoic acid), and that epithelial cells proliferate in response to vitamin A [31]. In addition to epithelial abnormalities, the VAD and VAD+VDD mice displayed abnormal immunoglobulin and cytokine/chemokine profiles compared to the controls. The conditions of VAD and VAD+VDD clearly rendered animals vulnerable to infection, morbidity, and mortality. Our results, in combination with previous reports, emphasize the importance of vitamin A in maintaining the physical integrity of epithelial barriers and in mounting effective immune responses to infections [32,33]. The findings encourage increased attention to vitamin A levels in the clinical arena of developed and developing countries. 

### 3.1. VAD, Keratinizing Metaplasia, UTIs and Respiratory Tract Disease

Our demonstrations of abnormalities in epithelial barriers supplement previous descriptions of poor health outcomes in the context of VAD. Children with severe VAD can suffer a chronic dry cough, increased susceptibility to respiratory infections, and pyuria, perhaps due to keratinizing metaplasia at mucosal surfaces [32,34,35,36,37]. Pathologies including UTIs and pyelonephritis have been previously described in Wistar rats and also in humans with VAD [32,38,39]. Acute pyelonephritis is a frequent cause of serious bacterial UTIs in infants, with renal scarring followed by hypertension and chronic renal failure being the most common long-term sequelae [40,41,42,43,44]. Vitamin A/retinoid supplementation can be protective in some (but not all) cases [39,45,46,47,48,49,50,51,52], perhaps in part by preventing the adhesion of bacteria to respiratory epithelium [33]. 

VAD has also been associated with the accelerated development of carcinogen-induced malignant neoplasms in the urinary tracts of rats. In one study, the administration of high-dose retinyl palmitate (≥250 IU/g diet), although unable to prevent the formation of transitional cell hyperplasia or neoplasia, prevented squamous metaplasia in animals receiving carcinogen [53].

In our virus study, the combination of VAD with infection was necessary for the development of squamous metaplasia in the lung. This result supplemented findings by Stephensen et. al., who observed squamous cell changes in the convalescent stage after VAD mice were infected with influenza virus [54]. Epithelial cell changes were also described in a study of cigarette smoke in VAD rats [55]. Abnormal squamous epithelium in terminal airways may compromise pulmonary defenses against infections by impeding the clearance of pathogens and cell debris from lower alveolar regions of the lung and upregulating mucosal cytokines [56]. 

Our findings with the SeV model may have additional significance given that squamous metaplasia of the bronchial epithelium is a pre-neoplastic change that develops in response to other airway insults, including inhaled irritants such as cigarette smoke [57,58,59]. Possibly, viral infections in VAD animals contribute to the development of pre-neoplastic changes in the lung that can, in some cases, progress to high-grade dysplasia and carcinoma [60,61]. The relationship between lung cancer and vitamin A precursors has been previously examined [62,63,64,65,66], and (as for other cancers) vitamin supplementation as a treatment for lung cancer has yielded variable results [67,68,69,70,71,72,73,74,75,76,77,78,79,80,81,82,83,84,85,86]. 

### 3.2. Dysregulation of Adaptive and Innate Immune Parameters in VAD Mice

We examined the immunoglobulin isotype profiles and cytokines/chemokines in the blood of animals on the four diets after PBS, CFA, or CFA/DNA/PEP injections. The “Combined VAD” mice exhibited differences in isotype profiles compared to the Non-VAD mice regardless of insult. The differences in the IgG2b/IgM ratios were highly significant and were most notable among males. This may be due to direct effects of vitamin A on class switch recombination and immunoglobulin expression [15,16,17,18], and/or to the indirect effects of T cells and cytokines on B cell development. An indication of T cell malfunction in VAD mice was the reduced level of RANTES (and IL-1α plus IL-5). RANTES and interleukins can be produced by T cells and can also regulate T cell activities [87,88,89]. The failed upregulation of RANTES in VAD animals following CFA/DNA/PEP stimuli helps to explain the poor recruitment of T cells to infected or otherwise damaged target tissues [90,91]. Unlike RANTES, IP-10 and eotaxin, typically produced by innate and barrier cells, were increased in the “Combined VAD” mice compared to Non-VAD mice, perhaps indicative of uncontrolled tissue damage. Abnormal immunoglobulin and cytokine patterns together mark immune dysfunction and set the stage for opportunistic infections [88,89,90,92,93,94,95]. 

### 3.3. Nuclear Receptors and Cross-Regulation

As stated above, vitamins and sex hormones have influences throughout the mammalian cell, but are best known for their binding of nuclear receptors and regulation of gene expression [11,14]. Each factor binds a set of receptors which serve as transcription factors, influential in promoter activity, enhancer activity, and chromosome structure. Our own work has identified response elements (sites to which nuclear receptors preferentially bind) for vitamins and estrogen in key regulatory regions, including switch sites of the immunoglobulin heavy chain locus [15]. One prominent hotspot for response elements appears in the position of the switch site Sμ, a site that is critical for the switch from IgM to other immunoglobulin isotypes. Additional response elements appear in or near promoters and enhancers of regulatory regions in the locus. Estrogen receptor binds these sites in activated B cells and shifts binding patterns (in concert with RNA polymerase) when B cells receive supplemental estrogen [16,17,96,97,98]. The removal of response elements in enhancer regions reduces the capacity for B cells to switch isotypes [96]. The response elements for vitamins and sex hormones overlap [15], providing an explanation for the immunoglobulin isotype differences between VAD females and males (Figure 1). Perhaps the receptors for vitamins and sex hormones cross-compete for binding to switch sites and regulatory elements, explaining why isotype patterns vary among male and female VAD animals. The ultimate composition of enhanceosomes and switchosomes may predict the patterns of CSR and resultant patterns of immunoglobulin isotype expression. The immunoglobulin expression patterns may in turn affect susceptibilities to infectious disease. We emphasize that mammalian cell types differ in their responses to nuclear factors [10], and that a composite of variables (diet, sex, genetics, and environment) will determine the host’s final outcome [96].

### 3.4. Defining Cause-Effect Relationships In Vivo

What are the precise cause-effect relationships among the parameters described in this report? We consider that in tissue culture settings, vitamin A directly and independently influences purified B cells and purified epithelial cells. However, parameter relationships are much more difficult to assess in vivo. A dampened immune response will render mice susceptible to infectious disease which will, in turn, damage epithelial cells. Damaged epithelial cells may increase infectious disease susceptibility and may drive immunopathological responses. These circular events are clearly detrimental to the VAD animal, particularly as the animal ages. The outcome is infection and death regardless of deliberate challenge or insult.

Of interest was the observation that squamous metaplasia in respiratory tissue was not apparent in young VAD mice, but became evident when the mice were infected with SeV. The results again portrayed complex interactions between nutritional status and pathogen invasions, in this case with an outcome of epithelial damage.

### 3.5. A focus on the Cross Regulatory Signals of Nuclear Receptors in Clinical Studies

The difficulty in interpreting clinical trial data is likely due, in part, to a limited focus on nuclear factors. For example, baseline vitamin levels are rarely measured among clinical trial participants. Our test of vitamin supplementation for the improvement of immune responses in healthy children demonstrated: (i) a strong correlation between baseline vitamin A and antibody responses and (ii) a benefit of vitamin supplements only when children were vitamin A and D-deficient or insufficient at baseline [99]. Our study suggested that vitamin A supplements may provide the greatest benefit if used in personalized medicine rather than with a one-size-fits-all treatment plan. One must also consider that vitamin A may convey both beneficial and harmful effects if used in patients with an acute infectious disease [100].

Despite its intricate relationship with pathogen control, vitamin A is often overlooked as a defense against clinical UTIs or respiratory diseases, particularly in developed countries [47,52]. Our results illustrate the dysfunctional immune parameters and poor outcomes of VAD and encourage clinicians to consider abnormal vitamin A levels as a cause of poor outcome. Programs that improve dietary intake among malnourished populations may correct immune functions, physical barriers, and protective mechanisms against infectious disease. 

## 4. Materials and Methods 

### 4.1. Animal Models

We followed the Association for Assessment and Accreditation for Laboratory Animal Care (AAALAC) guidelines. The protocols were approved by the Institutional Animal Care and Use Committee (IACUC) of St. Jude Children’s Research Hospital (Protocol #111, renewal 10 April 2018). To produce vitamin-deficient mice, pregnant female C57BL/6 mice were received from Jackson Laboratories (Bar Harbor, ME, USA) at day 4–5 gestation and immediately placed on test or control diets. The mice were fed characterized diets from Harlan Laboratories (Madison, WI, USA). The VAD diet was Harlan Cat# TD.10762. The VDD diet was Harlan Cat# TD.10763. The vitamin A+D double-deficient diet was Harlan Cat# TD.10616. The control diet included vitamin A palmitate (15 IU/g) and vitamin D (1.5 IU/g). This was Harlan Cat# TD.10764. Pups were maintained on the same diets as their mothers until adulthood (6–8 weeks of age, when experiments were begun) and throughout the course of the experiments. For animals depleted of vitamin D (VDD or VAD+VDD), cages were placed in dedicated cubicles with LED bulbs as the source of light to avoid UV-B irradiation. The animals were sacrificed if they were moribund during the course of the experiments. 

Retinol binding protein (RBP) tests and vitamin D tests were conducted to spot-check the vitamin levels in animals, as described previously [94]. RBP was used as a surrogate for retinol and was tested with an enzyme-linked immunosorbent assay (ELISA). The RBP tests confirmed that the VAD and VAD+VDD mice were all vitamin A-deficient (RBP was <5000 ng/mL), and there were significant differences in the RBP values when the VAD and VAD+VDD mice were compared to the VDD and control mice (unpaired *t*-test *p* < 0.0001, GraphPad Prism software, v8 [GraphPad Software, San Diego, CA, USA]). The vitamin D tests confirmed that the VDD and VAD+VDD mice were vitamin D-deficient (vitamin D was <15 nm/L), and there were significant differences in the vitamin D values when the VDD and VAD+VDD mice were compared to the VAD and control mice (unpaired *t*-test *p* < 0.0001). 

### 4.2. CFA or CFA/DNA/PEP Injections 

Animal groups were labeled CFA or CFA/DNA/PEP based on injection regimens. CFA: these animals received an IP priming dose with complete Freund’s adjuvant (Thermo Scientific, Waltham, MA, USA, Cat #77140, mixed 1:1 with PBS and emulsified) and one or two subsequent booster doses with incomplete Freund’s adjuvant (Thermo scientific Cat# 77145, mixed 1:1 with PBS and emulsified), administered at approximately three-week intervals. CFA/DNA/PEP: these animals received the same adjuvants and regimens described above, but adjuvants were mixed with Fus-1, a DNA-binding 27-mer peptide (KVCRRCYARLPVRASNCRKKACGHCSN; 10 µg per mouse) from *Trypanosoma cruzi*, which had been added dropwise to calf thymus DNA (Invitrogen #15633-019, 100 µg per mouse) prior to emulsification [22,23,24]. Additional animals received PBS only. Bleeds were taken 6 days to 4 weeks after the injections. The mice were sacrificed if they were moribund during the experimental course or after the final bleed. 

### 4.3. SeV infections

Young adult mice were briefly anesthetized with isoflurane and infected with SeV by intranasal inoculation (250–500 plaque forming units (pfu)). The mice were sacrificed after 22–29 days to evaluate their airway tissues. In some cases, the mice received a second dose of SeV (1.5 × 10^7^ pfu) two days before sacrifice.

### 4.4. Total Serum Immunoglobulin Analyses

The mouse sera, collected six days after the last booster injection, were diluted 1:25,000 and evaluated for multiple isotypes using the Luminex platform. 

The samples were run on a MILLIPLEX MAP mouse immunoglobulin isotyping kit (Millipore, Burlington, MA, USA, Cat# MGAMMAG-300K) and read on a Luminex 200 Multiplexing instrument using the xPonent software. The data were further processed using the Milliplex Analyst software. If the samples were below the lower limit of detection, they received a value of “0”. If the samples were above the upper limit of detection, they were assigned the upper limit value.

### 4.5. Histology

The mice were euthanized with isoflurane and the lungs were infused with formalin. The lungs and kidneys were then fixed by immersion in 10% neutral buffered formalin, embedded in paraffin, sectioned, and stained with hematoxylin and eosin stains. The slides were analyzed by a pathologist in the Veterinary Pathology Core Department of St. Jude Children’s Research Hospital. 

### 4.6. Cytokines

The sera were thawed, vortexed briefly, and spun at 2000 g for 1 min to pellet the debris. The sera were diluted 1:2 in an assay buffer and then assayed for Eotaxin, G-CSF, GM-CSF, interferon (IFN)γ, interleukin (IL)-1a, IL-1b, M-CSF, IL-2, IL-3, IL-4, IL-5, IL-6, IL-7, IL-10, IL-12(p40), IL-12(p70), IL-13, IL-15, IL-17, IFNγ-inducible protein (IP)-10, MIP-2, KC, LIF, LIX, MCP-1, MIP-1a, MIP-1b, MIG, regulated on activation, normal T cell expressed and secreted (RANTES), TNFα, VEGF, and IL-9 using a Mouse Cytokine/Chemokine Magnetic Bead Milliplex MAP kit (Millpore Cat# MCYTOMAG-70K-PX32). The plates were processed according to the manufacturer’s instructions and read using a Luminex 200 instrument. The results were evaluated using Excel and GraphPad Prism software, v8.

### 4.7. Statistical Analyses

Mann Whitney, Fisher’s Exact tests, and *t*-tests were performed using the GraphPad Prism software.

## Figures and Tables

**Table 1:** Frequent nephropathies in animals fed VAD or VAD+VDD diets.

Diets	Treatments						
	PBS		CFA		CFA/DNA/PEP		TOTAL
	Female	Male	Female	Male	Female	Male	All
***CONTROL***							
*Fraction*	0/8	0/11	1/10	0/13	0/11	0/9	1/62
*Percent*	0%	0%	10%	0%	0%	0%	2%
							
***VAD***							
*Fraction*	3/5	7/11	6/8	2/7	4/7	7/10	29/48
*Percent*	60%	64%	75%	29%	57%	70%	60%
							
***VDD***							
*Fraction*	1/12	0/12	0/10	0/14	0/7	0/14	1/69
*Percent*	8%	0%	0%	0%	0%	0%	1%
							
***VAD+VDD***							
*Fraction*	0/5	6/9	7/8	4/6	3/3	6/8	26/39
*Percent*	0%	67%	88%	67%	100%	75%	67%

Combined results from several experiments for which pathologies were analyzed demonstrated frequent severe kidney pathologies among the vitamin A deficient (VAD) and double deficient (VAD+VDD) animals compared to vitamin D deficient (VDD) and control animals. Female and male animals were examined at 3–6 months of age. We note that the experiments were not equally balanced for the numbers of animals per group.

## References

[1] Hussey G.D., Klein M. (1990). A randomized, controlled trial of vitamin A in children with severe measles. N. Engl. J. Med..

[2] Andersen A., Fisker A.B., Rodrigues A., Martins C., Ravn H., Lund N., Biering-Sorensen S., Benn C.S., Aaby P. (2018). National Immunization Campaigns with Oral Polio Vaccine Reduce All-Cause Mortality: A Natural Experiment within Seven Randomized Trials. Front. Public Health.

[3] (2013). Vitamin A supplementation: Who, when and how. Community Eye Health.

[4] Gilbert C. (2013). What is vitamin A and why do we need it?. Community Eye Health.

[5] Sommer A. (1993). Vitamin A, infectious disease, and childhood mortality: A 2 solution?. J. Infect. Dis..

[6] Jones B.G., Oshansky C.M., Bajracharya R., Tang L., Sun Y., Wong S.S., Webby R., Thomas P.G., Hurwitz J.L. (2016). Retinol binding protein and vitamin D associations with serum antibody isotypes, serum influenza virus-specific neutralizing activities and airway cytokine profiles. Clin. Exp. Immunol..

[7] Hurwitz J.L., Jones B.G., Penkert R.R., Gansebom S., Sun Y., Tang L., Bramley A.M., Jain S., McCullers J.A., Arnold S.R. (2017). Low Retinol-Binding Protein and Vitamin D Levels Are Associated with Severe Outcomes in Children Hospitalized with Lower Respiratory Tract Infection and Respiratory Syncytial Virus or Human Metapneumovirus Detection. J. Pediatr..

[8] Mora J.R., Iwata M., von Andrian U.H. (2008). Vitamin effects on the immune system: Vitamins A and D take centre stage. Nat. Rev. Immunol..

[9] Mora J.R., von Andrian U.H. (2004). Retinoic acid: An educational “vitamin elixir” for gut-seeking T cells. Immunity.

[10] Penkert R.R., Jones B.G., Hacker H., Partridge J.F., Hurwitz J.L. (2017). Vitamin A differentially regulates cytokine expression in respiratory epithelial and macrophage cell lines. Cytokine.

[11] Evans R.M., Mangelsdorf D.J. (2014). Nuclear Receptors, RXR, and the Big Bang. Cell.

[12] De Luca L.M. (1991). Retinoids and their receptors in differentiation, embryogenesis, and neoplasia. FASEB J..

[13] Shaw N., Elholm M., Noy N. (2003). Retinoic acid is a high affinity selective ligand for the peroxisome proliferator-activated receptor beta/delta. J. Biol. Chem..

[14] Napoli J.L. (2016). Functions of Intracellular Retinoid Binding-Proteins. Subcell Biochem..

[15] Hurwitz J.L., Penkert R.R., Xu B., Fan Y., Partridge J.F., Maul R.W., Gearhart P.J. (2016). Hotspots for Vitamin-Steroid-Thyroid Hormone Response Elements Within Switch Regions of Immunoglobulin Heavy Chain Loci Predict a Direct Influence of Vitamins and Hormones on B Cell Class Switch Recombination. Viral Immunol..

[16] Jones B.G., Sealy R.E., Penkert R.R., Surman S.L., Maul R.W., Neale G., Xu B., Gearhart P.J., Hurwitz J.L. (2018). Complex sex-biased antibody responses: Estrogen receptors bind estrogen response elements centered within immunoglobulin heavy chain gene enhancers. Int. Immunol..

[17] Jones B.G., Penkert R.R., Xu B., Fan Y., Neale G., Gearhart P.J., Hurwitz J.L. (2016). Binding of estrogen receptors to switch sites and regulatory elements in the immunoglobulin heavy chain locus of activated B cells suggests a direct influence of estrogen on antibody expression. Mol. Immunol..

[18] Sealy R.E., Jones B.G., Surman S.L., Penkert R.R., Pelletier S., Neale G., Hurwitz J.L. (2019). Will Attention by Vaccine Developers to the Host’s Nuclear Hormone Levels and Immunocompetence Improve Vaccine Success?. Vaccines (Basel).

[19] Stavnezer J., Schrader C.E. (2014). IgH chain class switch recombination: Mechanism and regulation. J. Immunol..

[20] Birshtein B.K. (2014). Epigenetic Regulation of Individual Modules of the immunoglobulin heavy chain locus 3′ Regulatory Region. Front. Immunol..

[21] Billiau A., Matthys P. (2001). Modes of action of Freund’s adjuvants in experimental models of autoimmune diseases. J. Leukoc. Biol..

[22] Desai D.D., Krishnan M.R., Swindle J.T., Marion T.N. (1993). Antigen-specific induction of antibodies against native mammalian DNA in nonautoimmune mice. J. Immunol..

[23] Desai D.D., Marion T.N. (2000). Induction of anti-DNA antibody with DNA-peptide complexes. Int. Immunol..

[24] Krishnan M.R., Wang C., Marion T.N. (2012). Anti-DNA autoantibodies initiate experimental lupus nephritis by binding directly to the glomerular basement membrane in mice. Kidney Int..

[25] Minano F.J., Fernandez-Alonso A., Benamar K., Myers R.K., Sancibrian M., Ruiz R.M., Armengol J.A. (1996). Macrophage inflammatory protein-1beta (MIP-1beta) produced endogenously in brain during E. coli fever in rats. Eur. J. Neurosci..

[26] Heslop R., Bojang A.L., Jarju S., Mendy J., Mulwa S., Secka O., Mendy F.S., Owolabi O., Kampmann B., Sutherland J.S. (2016). Changes in Host Cytokine Patterns of TB Patients with Different Bacterial Loads Detected Using 16S rRNA Analysis. PLoS ONE.

[27] Yamamoto Y., Klein T.W., Friedman H. (1996). Induction of cytokine granulocyte-macrophage colony-stimulating factor and chemokine macrophage inflammatory protein 2 mRNAs in macrophages by Legionella pneumophila or Salmonella typhimurium attachment requires different ligand-receptor systems. Infect. Immun..

[28] Allahverdian S., Harada N., Singhera G.K., Knight D.A., Dorscheid D.R. (2008). Secretion of IL-13 by airway epithelial cells enhances epithelial repair via HB-EGF. Am. J. Respir. Cell Mol. Biol..

[29] Bucco R.A., Zheng W.L., Davis J.T., Sierra-Rivera E., Osteen K.G., Chaudhary A.K., Ong D.E. (1997). Cellular retinoic acid-binding protein(II) presence in rat uterine epithelial cells correlates with their synthesis of retinoic acid. Biochemistry.

[30] Rudraraju R., Jones B.G., Surman S.L., Sealy R.E., Thomas P.G., Hurwitz J.L. (2014). Respiratory tract epithelial cells express retinaldehyde dehydrogenase ALDH1A and enhance IgA production by stimulated B cells in the presence of vitamin A. PLoS ONE.

[31] Nabeyrat E., Besnard V., Corroyer S., Cazals V., Clement A. (1998). Retinoic acid-induced proliferation of lung alveolar epithelial cells: Relation with the IGF system. Am. J. Physiol..

[32] Brown K.H., Gaffar A., Alamgir S.M. (1979). Xerophthalmia, protein-calorie malnutrition, and infections in children. J. Pediatr..

[33] Chandra R.K. (1988). Increased bacterial binding to respiratory epithelial cells in vitamin A deficiency. Bmj.

[34] Blackfan K.D., Wohlbach S.B. (1933). Vitamin A deficiency in infants: A clinical and pathological study. J. Pediatr..

[35] Pinnock C. (1991). Vitamin A. Nurs. J. India.

[36] Sommer A. (1983). Mortality associated with mild, untreated xerophthalmia. Trans. Am. Ophthalmol. Soc..

[37] Sommer A., Katz J., Tarwotjo I. (1984). Increased risk of respiratory disease and diarrhea in children with preexisting mild vitamin A deficiency. Am. J. Clin. Nutr..

[38] Bloch C.E. (1924). Further clinical investigations into the diseases arising in consequence of a deficiency in the fat-soluble A factor. Am. J. Dis. Child..

[39] Kavukcu S., Soylu A., Turkmen M., Sarioglu S., Buyukgebiz B., Gure A. (1999). The role of vitamin A in preventing renal scarring secondary to pyelonephritis. BJU Int..

[40] Shaikh N., Ewing A.L., Bhatnagar S., Hoberman A. (2010). Risk of renal scarring in children with a first urinary tract infection: A systematic review. Pediatrics.

[41] Neveus T. (2013). Can postpyelonephritic renal scarring be prevented?. Pediatr. Nephrol..

[42] Faust W.C., Diaz M., Pohl H.G. (2009). Incidence of post-pyelonephritic renal scarring: A meta-analysis of the dimercapto-succinic acid literature. J. Urol..

[43] Benador D., Benador N., Slosman D., Mermillod B., Girardin E. (1997). Are younger children at highest risk of renal sequelae after pyelonephritis?. Lancet.

[44] Jakobsson B., Berg U., Svensson L. (1994). Renal scarring after acute pyelonephritis. Arch. Dis. Child..

[45] Kavukcu S., Turkmen M., Sevinc N., Soylu A., Derebek E., Buyukgebiz B. (1998). Serum vitamin A and beta-carotene concentrations and renal scarring in urinary tract infections. Arch. Dis. Child..

[46] Kavukcu S., Turkmen M.A., Soylu A. (2001). Could the effective mechanisms of retinoids on nephrogenesis be also operative on the amelioration of injury in acquired renal lesions?. Pediatr. Nephrol..

[47] Yilmaz A., Bahat E., Yilmaz G.G., Hasanoglu A., Akman S., Guven A.G. (2007). Adjuvant effect of vitamin A on recurrent lower urinary tract infections. Pediatr. Int..

[48] Wagner J. (2001). Potential role of retinoids in the therapy of renal disease. Nephrol. Dial. Transplant..

[49] Sobouti B., Hooman N., Movahed M. (2013). The effect of vitamin E or vitamin A on the prevention of renal scarring in children with acute pyelonephritis. Pediatr. Nephrol..

[50] Bennett R.T., Mazzaccaro R.J., Chopra N., Melman A., Franco I. (1999). Suppression of renal inflammation with vitamins A and E in ascending pyelonephritis in rats. J. Urol..

[51] Ayazi P., Moshiri S.A., Mahyar A., Moradi M. (2011). The effect of vitamin A on renal damage following acute pyelonephritis in children. Eur. J. Pediatr..

[52] Flores-Mireles A.L., Walker J.N., Caparon M., Hultgren S.J. (2015). Urinary tract infections: Epidemiology, mechanisms of infection and treatment options. Nat. Rev. Microbiol..

[53] Cohen S.M., Wittenberg J.F., Bryan G.T. (1976). Effect of avitaminosis A and hypervitaminosis A on urinary bladder carcinogenicity of N-(4-(5-Nitro-2-furyl)-2-thiazolyl)formamide. Cancer Res..

[54] Stephensen C.B., Blount S.R., Schoeb T.R., Park J.Y. (1993). Vitamin A deficiency impairs some aspects of the host response to influenza A virus infection in BALB/c mice. J. Nutr..

[55] Shields P.A., Jeffery P.K. (1987). The combined effects of vitamin A-deficiency and cigarette smoke on rat tracheal epithelium. Br. J. Exp. Pathol..

[56] Penkert R.R., Surman S.L., Jones B.G., Sealy R.E., Vogel P., Neale G., Hurwitz J.L. (2016). Vitamin A deficient mice exhibit increased viral antigens and enhanced cytokine/chemokine production in nasal tissues following respiratory virus infection despite the presence of FoxP3+ T cells. Int. Immunol..

[57] Barsky S.H., Roth M.D., Kleerup E.C., Simmons M., Tashkin D.P. (1998). Histopathologic and molecular alterations in bronchial epithelium in habitual smokers of marijuana, cocaine, and/or tobacco. J. Natl. Cancer Inst..

[58] Mathe G., Gouveia J., Hercend T., Gros F., Dorval T., Hazon J., Misset J.L., Schwarzenberg L., Ribaud P., Lemaigre G. (1982). Correlation between precancerous bronchial metaplasia and cigarette consumption, and preliminary results of retinoid treatment. Cancer Detect. Prev..

[59] Peters E.J., Morice R., Benner S.E., Lippman S., Lukeman J., Lee J.S., Ro J.Y., Hong W.K. (1993). Squamous metaplasia of the bronchial mucosa and its relationship to smoking. Chest.

[60] Wistuba I.I., Behrens C., Milchgrub S., Bryant D., Hung J., Minna J.D., Gazdar A.F. (1999). Sequential molecular abnormalities are involved in the multistage development of squamous cell lung carcinoma. Oncogene.

[61] Giroux V., Rustgi A.K. (2017). Metaplasia: Tissue injury adaptation and a precursor to the dysplasia-cancer sequence. Nat. Rev. Cancer.

[62] Sun S.Y., Lotan R. (2002). Retinoids and their receptors in cancer development and chemoprevention. Crit. Rev. Oncol. Hemat..

[63] Wald N., Idle M., Boreham J., Bailey A. (1980). Low serum-vitamin-A and subsequent risk of cancer. Preliminary results of a prospective study. Lancet.

[64] Willett W.C. (1990). Vitamin A and lung cancer. Nutr. Rev..

[65] Shareck M., Rousseau M.C., Koushik A., Siemiatycki J., Parent M.E. (2017). Inverse Association between Dietary Intake of Selected Carotenoids and Vitamin C and Risk of Lung Cancer. Front. Oncol..

[66] Yu N., Su X., Wang Z., Dai B., Kang J. (2015). Association of Dietary Vitamin A and beta-Carotene Intake with the Risk of Lung Cancer: A Meta-Analysis of 19 Publications. Nutrients.

[67] Bukhari M.H., Qureshi S.S., Niazi S., Asef M., Naheed M., Khan S.A., Chaudhry N.A., Tayyab M., Hasan M. (2007). Chemotherapeutic/chemopreventive role of retinoids in chemically induced skin carcinogenesis in albino mice. Int. J. Dermatol..

[68] Li Y., Zhang Y., Hill J., Kim H.T., Shen Q., Bissonnette R.P., Lamph W.W., Brown P.H. (2008). The rexinoid, bexarotene, prevents the development of premalignant lesions in MMTV-erbB2 mice. Br. J. Cancer.

[69] Moon R.C., Kelloff G.J., Detrisac C.J., Steele V.E., Thomas C.F., Sigman C.C. (1994). Chemoprevention of OH-BBN-induced bladder cancer in mice by oltipraz, alone and in combination with 4-HPR and DFMO. Anticancer Res..

[70] Pisano C., Vesci L., Fodera R., Ferrara F.F., Rossi C., De Cesare M., Zuco V., Pratesi G., Supino R., Zunino F. (2007). Antitumor activity of the combination of synthetic retinoid ST1926 and cisplatin in ovarian carcinoma models. Ann. Oncol..

[71] Shah R.K., Valdez T.A., Wang Z., Shapshay S.M. (2001). Pulsed-dye laser and retinoic acid delay progression of oral squamous cell carcinoma: A murine model. Laryngoscope.

[72] Wang Y., Wen W., Yi Y., Zhang Z., Lubet R.A., You M. (2009). Preventive effects of bexarotene and budesonide in a genetically engineered mouse model of small cell lung cancer. Cancer Prev. Res. (Phila.).

[73] Edelman M.J., Smith R., Hausner P., Doyle L.A., Kalra K., Kendall J., Bedor M., Bisaccia S. (2005). Phase II trial of the novel retinoid, bexarotene, and gemcitabine plus carboplatin in advanced non-small-cell lung cancer. J. Clin. Oncol..

[74] Peterlin P., Garnier A., Tissot A., Garandeau C., Houreau-Langlard D., Hourmant M., Vantyghem S., Bonnet A., Guillaume T., Bene M.C. (2016). Successful treatment of acute promyelocytic leukemia with arsenic trioxide and all-trans retinoic acid in a double lung and kidney transplanted patient. Ann. Hematol..

[75] Recchia F., Sica G., Candeloro G., Necozione S., Bisegna R., Bratta M., Rea S. (2009). Beta-interferon, retinoids and ta/moxifen in metastatic breast cancer: Long-term follow-up of a phase II study. Oncol. Rep..

[76] Colombo N., Formelli F., Cantu M.G., Parma G., Gasco M., Argusti A., Santinelli A., Montironi R., Cavadini E., Baglietto L. (2006). A phase I-II preoperative biomarker trial of fenretinide in ascitic ovarian cancer. Cancer Epidemiol. Biomark..

[77] Alpha-Tocopherol, Beta Carotene Cancer Prevention Study Group (1994). The effect of vitamin E and beta carotene on the incidence of lung cancer and other cancers in male smokers. N. Engl. J. Med..

[78] De Vries N., Van Zandwijk N., Pastorino U. (1993). The EUROSCAN study: A progress report. Am. J. Otolaryngol..

[79] Hennekens C.H., Buring J.E., Manson J.E., Stampfer M., Rosner B., Cook N.R., Belanger C., LaMotte F., Gaziano J.M., Ridker P.M. (1996). Lack of effect of long-term supplementation with beta carotene on the incidence of malignant neoplasms and cardiovascular disease. N. Engl. J. Med..

[80] Omenn G.S. (2007). Chemoprevention of lung cancers: Lessons from CARET, the beta-carotene and retinol efficacy trial, and prospects for the future. Eur. J. Cancer Prev..

[81] Satia J.A., Littman A., Slatore C.G., Galanko J.A., White E. (2009). Long-term use of beta-carotene, retinol, lycopene, and lutein supplements and lung cancer risk: Results from the VITamins And Lifestyle (VITAL) study. Am. J. Epidemiol..

[82] Omenn G.S., Goodman G.E., Thornquist M.D., Balmes J., Cullen M.R., Glass A., Keogh J.P., Meyskens F.L., Valanis B., Williams J.H. (1996). Effects of a combination of beta carotene and vitamin A on lung cancer and cardiovascular disease. N. Engl. J. Med..

[83] Martinez M.E., Jacobs E.T., Baron J.A., Marshall J.R., Byers T. (2012). Dietary supplements and cancer prevention: Balancing potential benefits against proven harms. J. Natl. Cancer Inst..

[84] Druesne-Pecollo N., Latino-Martel P., Norat T., Barrandon E., Bertrais S., Galan P., Hercberg S. (2010). Beta-carotene supplementation and cancer risk: A systematic review and metaanalysis of randomized controlled trials. Int. J. Cancer.

[85] Neuhouser M.L., Patterson R.E., Thornquist M.D., Omenn G.S., King I.B., Goodman G.E. (2003). Fruits and vegetables are associated with lower lung cancer risk only in the placebo arm of the beta-carotene and retinol efficacy trial (CARET). Cancer Epidemiol. Biomark. Prev..

[86] Russell R.M. (2004). The enigma of beta-carotene in carcinogenesis: What can be learned from animal studies. J. Nutr..

[87] Cerdan C., Martin Y., Brailly H., Courcoul M., Flavetta S., Costello R., Mawas C., Birg F., Olive D. (1991). IL-1 alpha is produced by T lymphocytes activated via the CD2 plus CD28 pathways. J. Immunol..

[88] Culley F.J., Pennycook A.M., Tregoning J.S., Dodd J.S., Walzl G., Wells T.N., Hussell T., Openshaw P.J. (2006). Role of CCL5 (RANTES) in viral lung disease. J. Virol..

[89] Rammal A., Tewfik M., Rousseau S. (2017). Differences in RANTES and IL-6 levels among chronic rhinosinusitis patients with predominant gram-negative and gram-positive infection. J. Otolaryngol. Head Neck Surg..

[90] Rudraraju R., Surman S.L., Jones B.G., Sealy R., Woodland D.L., Hurwitz J.L. (2012). Reduced frequencies and heightened CD103 expression among virus-induced CD8(+) T cells in the respiratory tract airways of vitamin A-deficient mice. Clin. Vaccine Immunol..

[91] Crawford A., Angelosanto J.M., Nadwodny K.L., Blackburn S.D., Wherry E.J. (2011). A role for the chemokine RANTES in regulating CD8 T cell responses during chronic viral infection. PLoS Pathog..

[92] Surman S.L., Jones B.G., Rudraraju R., Sealy R.E., Hurwitz J.L. (2014). Intranasal administration of retinyl palmitate with a respiratory virus vaccine corrects impaired mucosal IgA response in the vitamin A-deficient host. Clin. Vaccine Immunol..

[93] Surman S.L., Jones B.G., Sealy R.E., Rudraraju R., Hurwitz J.L. (2014). Oral retinyl palmitate or retinoic acid corrects mucosal IgA responses toward an intranasal influenza virus vaccine in vitamin A deficient mice. Vaccine.

[94] Surman S.L., Penkert R.R., Jones B.G., Sealy R.E., Hurwitz J.L. (2016). Vitamin supplementation at the time of immunization with cold-adapted influenza virus vaccine corrects poor antibody responses in mice deficient for vitamins A and D. Clin. Vaccine Immunol..

[95] Surman S.L., Rudraraju R., Sealy R., Jones B., Hurwitz J.L. (2012). Vitamin A deficiency disrupts vaccine-induced antibody-forming cells and the balance of IgA/IgG isotypes in the upper and lower respiratory tract. Viral Immunol..

[96] Jones B.G., Penkert R.R., Surman S.L., Sealy R.E., Pelletier S., Berns H., Hurwitz J.L. (2019). Background check: Profound differences in serum antibody isotypes among C57BL/6 mouse substrains discourage substrain interchanges in immunology experiments. Immunol. Lett..

[97] Jones B.G., Penkert R.R., Surman S.L., Sealy R.E., Pelletier S., Xu B., Neale G., Maul R.W., Gearhart P.J., Hurwitz J.L. (2019). Matters of life and death: How estrogen and estrogen receptor binding to the immunoglobulin heavy chain locus may influence outcomes of infection, allergy, and autoimmune disease. Cell. Immunol..

[98] Jones B.G., Sealy R.E., Penkert R.R., Surman S.L., Birshtein B.K., Xu B., Neale G., Maul R.W., Gearhart P.J., Hurwitz J.L. (2020). From Influenza Virus Infections to Lupus: Synchronous Estrogen Receptor alpha and RNA Polymerase II Binding Within the Immunoglobulin Heavy Chain Locus. Viral Immunol..

[99] Patel N., Penkert R.R., Jones B.G., Sealy R.E., Surman S.L., Sun Y., Tang L., DeBeauchamp J., Webb A., Richardson J. (2019). Baseline Serum Vitamin A and D Levels Determine Benefit of Oral Vitamin A&D Supplements to Humoral Immune Responses Following Pediatric Influenza Vaccination. Viruses.

[100] Bresee J.S., Fischer M., Dowell S.F., Johnston B.D., Biggs V.M., Levine R.S., Lingappa J.R., Keyserling H.L., Petersen K.M., Bak J.R. (1996). Vitamin A therapy for children with respiratory syncytial virus infection: A multicenter trial in the United States. Pediatr. Infect. Dis. J..

